# Cerebrospinal fluid lactate levels along the Alzheimer’s disease continuum and associations with blood-brain barrier integrity, age, cognition, and biomarkers

**DOI:** 10.1186/s13195-022-01004-9

**Published:** 2022-04-26

**Authors:** Paul Theo Zebhauser, Achim Berthele, Oliver Goldhardt, Janine Diehl-Schmid, Josef Priller, Marion Ortner, Timo Grimmer

**Affiliations:** 1 Department of Neurology, School of Medicine, Technical University of Munich, Munich, Germany; 2 Department of Psychiatry and Psychotherapy, School of Medicine, Technical University of Munich, Munich, Germany; 3 Charité-Universitätsmedizin Berlin and DZNE, Berlin, Germany; 4 University of Edinburgh and UK DRI, Edinburgh, UK

**Keywords:** Alzheimer’s disease, Cerebrospinal fluid, Lactate, Biomarkers, Blood-brain barrier

## Abstract

**Background:**

Cerebrospinal fluid (CSF) lactate levels have been suggested to be associated with disease severity and progression in several neurological diseases as an indicator of impaired energy metabolism, neuronal death, or microglial activation. Few studies have examined CSF lactate levels in dementia due to Alzheimer’s disease (AD) and found higher values in AD patients compared to healthy controls (HC). However, these studies were mostly small in size, the inclusion criteria were not always well defined, and the diagnostic value and pathophysiological significance of CSF lactate in AD remain unclear.

**Methods:**

We examined CSF lactate levels and potentially associated factors in a large (*n*=312), biologically and clinically well-defined sample of patients with AD at the stage of mild cognitive impairment (MCI-AD) and dementia (ADD), HC, and patients with frontotemporal lobar degeneration (FTLD).

**Results:**

Contrary to previous studies, patients with ADD and HC did not differ in CSF lactate levels. However, we found higher values for patients with MCI-AD compared to those with ADD and to HC in univariate analysis, as well as for MCI-AD compared to ADD when controlling for age and blood-brain barrier integrity. CSF lactate levels were associated with age and blood-brain barrier integrity but not with clinical severity or CSF biomarkers of AD.

**Conclusions:**

CSF lactate does not indicate biological or clinical disease severity in AD, nor does it differentiate between patients with AD and HC or patients with FTLD. However, higher CSF lactate levels were found in earlier stages of AD, which might be interpreted in the context of inflammatory processes.

## Introduction/background

Metabolic dysfunction of the central nervous system (CNS) has been discussed as a pathophysiological key contributor in Alzheimer’s disease (AD). Evidence from animal and in vitro studies points to disturbances in glucose transport mechanisms, disrupted glycolysis, oxidative stress and impaired mitochondrial function [1], which is mirrored by alterations in glucose metabolism in PET studies of the AD brain [2]. In recent years, ketogenic diet has even been proposed as a treatment option for Alzheimer’s disease dementia (ADD) by potentially ramping up energy metabolism in the diseased brain [3].

Lactate in the CNS has traditionally been interpreted as a metabolic waste product, while in recent years it has been found to serve a wide range of physiological functions from modulating neuronal excitability to contributing to energy homeostasis [4]. However, markedly elevated lactate levels in rodent brain tissue have been found and discussed as a feature of aging [5] and a certain age-dependency of lactate concentrations has been observed for human CSF [6]. In multiple sclerosis, CSF lactate has been reported to correlate weakly to moderately with disease severity and progression [7, 8]. In mitochondrial diseases [9] and hepatic encephalopathy [10], elevated CSF lactate has been interpreted as a marker of impaired energy metabolism.

Impaired mitochondrial functioning and glycolysis pathways have been found in AD [1] and a small number of studies investigated CSF lactate levels in patients with ADD. Redjems-Bennani et al. [11] found higher CSF lactate levels in 17 patients compared to 17 controls. Similar results in marginally larger samples were found by Parnetti and colleagues in two separate studies [12, 13]. A more recent study with a sample size of over 200 patients and controls [14] confirmed higher CSF lactate concentrations in AD patients. Interestingly, the authors found higher lactate levels in patients with less severe cognitive impairment. Also, a strong negative correlation with tau-proteins in the CSF was observed. Opposed to these results, one small sample-sized study found lower CSF-lactate concentrations in patients with ADD compared to healthy controls [15].

Taken together, previous work suggested elevated CSF lactate levels in ADD, but most of the studies were limited by small sample sizes. Furthermore, limited characterization of ADD patients and control subjects poses a major problem in these studies. Thus, in none of the studies was the inclusion of patients based on distinct cut-off values for core biomarkers of AD-like amyloid-beta 1–42 (Aβ42) or tau-proteins. In the meantime, this has become the gold standard for studies in the field of AD, which is reflected by recently published research criteria for AD [16]. Regarding healthy controls, only one study included volunteers without any neurological symptoms [15]. In the remaining studies, subjects underwent lumbar puncture for diagnostic purposes (e.g., ruling out subarachnoid hemorrhage in headache patients) or the reason for CSF collection was not specified further. Furthermore, levels of CSF lactate have not yet been compared between patients with AD at the stage of mild cognitive impairment (MCI) and at the stage of dementia. This would be of importance when investigating lactate concentrations as a possible measure of disturbed brain metabolism in the context of AD, as one would expect an association of concentrations with disease severity. Moreover, the integrity of the blood-brain barrier (BBB) might influence CSF lactate levels, which has not been considered in previous studies. While normally lactate crosses the BBB via monocarboxylate carriers, disturbances of BBB function might affect regulatory mechanisms of lactate efflux and influx. Progressive dysfunction of the BBB is a characteristic of the aging brain [17] and a possible feature of AD [18, 19].

In recent years, neuroinflammation and microglial activation have been discussed as key elements in the pathogenesis of AD [20]. In this context, it is crucial to note that elevated lactate levels could be indicative not only of changes in energy metabolism, but also of inflammatory events and processes. Along these lines, the infection hypothesis of AD, which postulates a causative role of e.g. herpes viruses in the pathophysiological development of AD [21, 22] should be mentioned, as latent infectious processes in the CNS could cause elevated CSF lactate levels in patients with AD.

In summary, it is still unclear if CSF lactate concentrations differ between patients with AD and healthy controls. Moreover, data regarding associations of CSF lactate levels with clinical and biological proxies for AD disease severity are still missing.

In this study, we compared CSF lactate levels in a large and well-defined sample of patients with AD dementia, MCI due to AD and healthy controls under consideration of potentially associated or even confounding factors like BBB integrity, age, and proxies of disease severity (CSF biomarkers of AD and cognitive measures). For comparison, we included a subset of patients suffering from dementia with frontotemporal lobar degeneration (FTLD).

## Methods

Data was extracted from the CSF biobank of the Department of Psychiatry and Psychotherapy, Klinikum rechts der Isar, Technical University of Munich. All study participants provided written informed consent.

### Sample characteristics and study procedures

We analyzed CSF data from patients with ADD, MCI-AD, and FTLD. As part of clinical routine procedures, CSF lactate levels and Albumin CSF/serum ratios (Alb CSF/S) were assessed for differential diagnosis. As a control group, CSF samples from healthy volunteers were acquired in the context of spinal anesthesia prior to orthopedic or urologic surgery procedures. Inclusion criteria were the absence of any neurological or psychiatric diseases (as indicated by clinical examination, medical history and review of patient files), absence of subjective cognitive impairment, and intact global cognitive functioning defined by Mini-Mental State examination scores (MMSE) [23]; of at least 28 points [24]. Inclusion criteria for the subsamples are summarized in Table 1.

**Table 1:** Inclusion criteria for subsamples

	ADD	MCI-AD	FTLD	HC
**CSF Aβ42**	<650 pg/nl	<650 pg/nl	-	>649 pg/nl
**MMSE**	<25	>24	-	>27
**CSF lactate**	<3.5mmol/l	<3.5mmol/l	<3.5mmol/l	<3.5mmol/l
**Albumin CSF/serum**	<15	<15	<15	<15
**Diagnostic criteria**	McKhann et al. (29)	Albert et al. (2011)	Rascovsky et al. (2011) Gorno-Tempini et al. (2011)	No subjective cognitive impairment; no neurological/psychiatric disease^a^

^a^Indicated by clinical examination, medical history, and review of patient files

*ADD* Alzheimer’s disease dementia, *MCI-AD* mild cognitive impairment due to Alzheimer’s disease, *FTLD* frontotemporal lobar degeneration, *HC* healthy controls

Diagnoses of ADD, MCI-AD and FTLD were made by board-certified psychiatrists and experts in the field of dementia and aging in accordance with internationally recognized consensus criteria. For ADD, the criteria by McKhann et al. (2011) were used. For being classified as MCI-AD, we used the general criteria by Albert et al. [25]. In this context, cognitive impairment (defined as ≤1.5 standard deviations below the sex-, age- and education-adjusted mean in at least one cognitive test) was evaluated using Consortium to Establish a Registry for Alzheimer's Disease neuropsychological battery (CERAD-N)B [26];, an extensive neuropsychological testing battery covering the domains of complex attention, executive function, learning and memory, language, and perceptual–motor function. For diagnosing FLTD, i.e. behavioral variant frontotemporal dementia, semantic variant and non-fluent variant of primary progressive aphasia, the criteria established by Rascovsky et al. [27] and Gorno-Tempini et al. [28] were used. Furthermore, we only included ADD patients with MMSE scores < 25 and MCI-AD patients with MMSE scores > 24 points. In line with recent research guidelines for AD, we only included patients with ADD and MCI-AD with a positive amyloid biomarker profile according to the A/T/N framework proposed by the National Institute on Aging and Alzheimer's Association [16]. For this purpose, we used a CSF Aβ42 cut-off value of < 650 pg/ml (cut-off-value established in-house using amyloid PET positivity as standard of truth in an independent cohort). Consequently, we excluded healthy participants with abnormal Aβ42 concentrations to rule out asymptomatic AD. In all patients and controls, we also examined CSF total-Tau (t-Tau) levels as a general measure of neurodegeneration. As we aimed to characterize patients along the AD continuum (MCI-AD and ADD) and t-Tau can be considered a downstream marker of AD-pathology [29], we did not apply an in- or exclusion criterion for CSF total Tau (t-Tau).

To rule out loss of BBB integrity due to additional undiagnosed underlying nervous system pathology (e.g., severe radiculopathy or tumor) we excluded patients with an Albumin CSF to serum ratio (Alb CSF/S) greater than 15. The rationale behind that was that we examined the integrity of the BBB as a possible influencing factor on CSF lactate levels. Additionally, we excluded patients with CSF lactate concentrations > 3.5 mmol/l due to possible acute infectious central nervous processes.

### CSF collection and analysis

For CSF collection into polypropylene tubes, lumbar puncture was performed between segments L3/4 or L4/5. All samples were immediately stored on ice in upright position and processed within 1 hour. CSF lactate levels and albumin levels in CSF and serum were measured following clinical standard procedures. CSF for peptide concentrations (t-Tau, Aβ42) was centrifuged at 2000×*g* and 4°C for 10 min, and aliquots were stored at – 80 °C. Peptide concentrations were analyzed with commercially available enzyme-linked immunosorbent assays (ELISA; INNOTEST hTAU Ag, Fujirebio Europe N.V.; IBL International GmbH; Amyloid-Beta [1-42] CSF ELISA, IBL International GmbH) following standardized procedures in our round robin test-certified laboratory for neurochemical analyses.

### Statistical analysis

Data was analyzed using *R: A language and environment for statistical computing* [30], *Version 3.5.3.* Normality was checked for by employing Shapiro-Wilk-tests and inspection of histograms. As most continuous variables were distributed non-normally, predominantly non-parametric methods were applied.

To explore the relationships of CSF lactate levels with t-Tau and Aβ42 concentrations, Alb CSF/S, age, and MMSE scores, Spearman rank correlations were calculated. Significance levels were alpha adjusted using the Bonferroni correction. For univariate between-group analyses for CSF lactate levels, a Kruskal-Wallis test was performed. In a second step, multivariate regression models to control for potential confounders were carried out. Categorical predictors (group level) were dummy-coded. Continuous predictors/potential confounders were selected based on correlation analyses; all independent variables were entered into the model simultaneously.

## Results

### Sample characteristics

A total number of *n*=312 patients and volunteers met the delineated inclusion criteria after excluding *n*=5 patients for severe disruption of BBB integrity (*n*=4 ADD, *n*=1 MCI-AD), *n*=2 patients for showing severely elevated CSF lactate levels (*n*=1 MCI-AD, *n*=1 HC), and *n*=12 healthy volunteers for CSF-amyloid-positivity.

### Descriptive statistics

Table 2 depicts descriptive values of CSF variables, demographics, and MMSE scores for patients with ADD, MCI-AD, FTLD, and HC. Note, that the proportion of female participants was slightly higher in ADD and FTLD patients, and slightly lower in HC. However, Mann-Whitney *U* tests revealed no significant differences in CSF lactate levels (as the primary variable of interest) between sexes on single group levels *(p* = 0.539 for ADD, *p* = 0.463 for MCI-AD, *p* = 0.080 for FTLD and *p* = 0.097 for HC), nor in a pooled analysis of all samples (*p* = 0.147).

**Table 2:** Descriptive values of demographics, MMSE scores, and CSF analyses

		ADD	MCI-AD	FTLD	HC
***n*** **(%f)**		119 (59.7)	101 (47.5)	34 (58.8)	58 (34.5)
**Age [years]**	*M (SD)*	66.4 (9.5)	68.4 (10.0)	66.2 (8.6)	59.1 (13.7)
	*MD (IQR)*	67 (60–73)	72 (82–76)	64.0 (59.3–74.0)	60.5 (51.0–70.8)
	*Range*	40–89	41–84	46–83	30–85
**MMSE [points]**	*M (SD)*	19.5 (4.8)	26.8 (1.3)	20.5 (6.6)	29.5 (0.7)
	*MD (IQR)*	21 (18–23)	27 (60–28)	22.5 (16.3–26.0)	30.0 (29.0–30.0)
	*Range*	1–24	25–30	2–30	28–30
**CSF lactate [mmol/l]**	*M (SD)*	1.75 (0.25)	1.83 (0.24)	1.79 (0.23)	1.73 (0.31)
	*MD (IQR)*	1.72 (1.60–1.90)	1.80 (1.69–1.95)	1.80 (1.63–1.93)	1.69 (1.50–1.90)
	*Range*	1.20–1.70	1.30–2.60	1.30–2–27	1.10–2.70
**Albumin CSF/serum**	*M (SD)*	6.82 (2.63)	6.61 (2.56)	6.23 (2.19)	6.91 (2.92)
	*MD (IQR)*	6.38 (4.87–8.22)	5.88 (4.80–7.56)	5.81 (4.78–6.80)	5.94 (4.83–8.32)
	*Range*	2.55–14.40	2.98–13.20	3.32–13.10	2.68–14.30
**CSF Aβ42 [pg/nl]**	*M (SD)*	464 (116)	452 (110)	662 (438)	1029 (252)
	*MD (IQR)*	471 (393–552)	469 (368–551)	662 (470–975)	1029 (816–1219)
	*Range*	226–649	182–638)	334–2352	670–1714
**CSF t-Tau [pg/nl]**	*M (SD)*	797 (638)	554 (360)	685 (706)	262 (101)
	*MD (IQR)*	630 (397–991)	461 (342–680)	451 (331–659)	246 (183–314)
	*Range*	122–4635	37–1708	70–3600	108–551

*n* number, *f* female, *m* male, *M* mean, *SD* standard deviation, *MD* median, *IQR* interquartile range, *ADD* Alzheimer’s disease dementia, *MCI-AD* mild cognitive impairment due to Alzheimer’s disease, *FTLD* frontotemporal lobar degeneration, *HC* healthy controls

### Correlation analyses

**Fig. 1:**
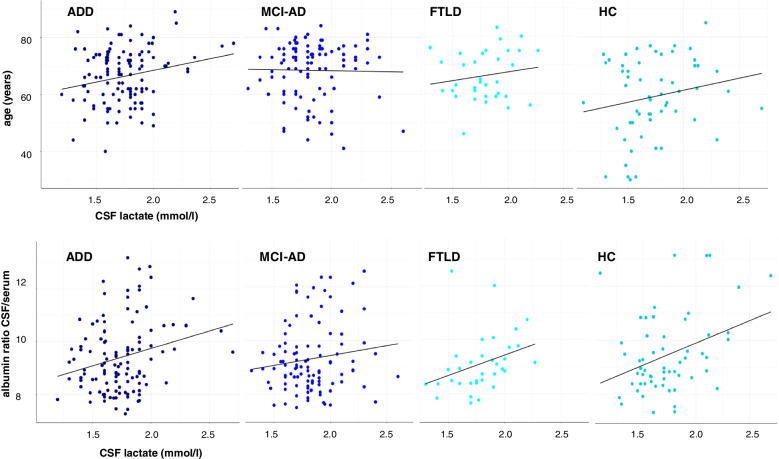
Scatterplots for correlations of CSF lactate concentration with age and Alb CSF/S. Note: ADD, Alzheimer’s disease dementia; MCI AD, mild cognitive impairment due to Alzheimer’s disease; FTLD, frontotemporal lobar degeneration; HC, healthy controls. Identical units apply for *x*-axis in each plot

Table 3 shows Spearman rank correlation coefficients of variables of interest with CSF lactate concentrations on a single-group level for patients with ADD, MCI-AD, FTLD, and HC, as well as for pooled data across groups. On a single group level, Alb CSF/S was correlated with lactate concentrations for patients with ADD and FTLD and for HC. Statistical significance remained after Bonferroni correction for comparisons on group levels only for FTLD patients. For HC, lactate concentrations were associated with t-Tau concentrations before adjusting alpha levels for comparisons on the group level. In a pooled correlation analysis for all groups, age and Alb CSF/S was correlated with lactate concentrations, both still significant after Bonferroni correction for comparisons on a group level, the latter even after correction across groups. In Fig. 1, scatter plots for correlations of lactate concentrations with age and Alb CSF/S are shown. As variability in Alb CSF/S was considerably higher than in CSF lactate levels across groups (see Table 2), we additionally carried out correlation analyses for those variables with a more rigid exclusion criterion for BBB integrity (Alb CSF/S >10). Correlations coefficients were comparable for FTLD (*r* = 0.598, *p* < 0.001), HC (*r* = 0.262, *p* = 0.061) and decreased for MCI-AD (*r* = − 0.093, *p* = 0.392) and ADD (*r* = 0.090, *p* = 0.362). However, testing for differences in correlation coefficients [31] resulted in non-significant differences (all *p*’s > 0.1).

**Table 3:** Correlations of CSF lactate with variables of interest for subsamples

	ADD	MCI-AD	FTLD	HC	all
**Age [years]**	0.133	0.028	0.150	0.179	0.156**
	*p = 0.148*	*p = 0.782*	*p = 0.396*	*p = 0.179*	***p = 0.006***
**MMSE [points]**	− 0.054	0.019	− 0.208	0.035	− 0.007
	*p = 0.562*	*p = 0.851*	*p = 0.238*	*p = 0.794*	*p = 0.905*
**Albumin CSF/serum**	0.216*	0.081	0.501**	0.302*	0.207***
	***p = 0.018***	*p = 0.422*	***p = 0.003***	***p = 0.021***	***p < 0.001***
**CSF Aβ42 [pg/nl]**	− 0.143	− 0.023	0.066	− 0.008	− 0.104
	*p = 0.121*	*p = 0.819*	*p = 0.712*	*p = 0.951*	*p = 0.067*
**CSF t-Tau [pg/nl]**	− 0.030	− 0.064	0.032	0.275*	0.070
	*p = 0.749*	*p = 0.525*	*p = 0.859*	***p = 0.036***	*p = 0.220*

*Significant at *p*<0.05

**Significant after Bonferroni correction on group level (*p*<0.05/5= <0.01)

***Significant after Bonferroni correction across groups (*p*<0.05/25= <0.002)

### Between-group differences in lactate concentrations

**Fig. 2:**
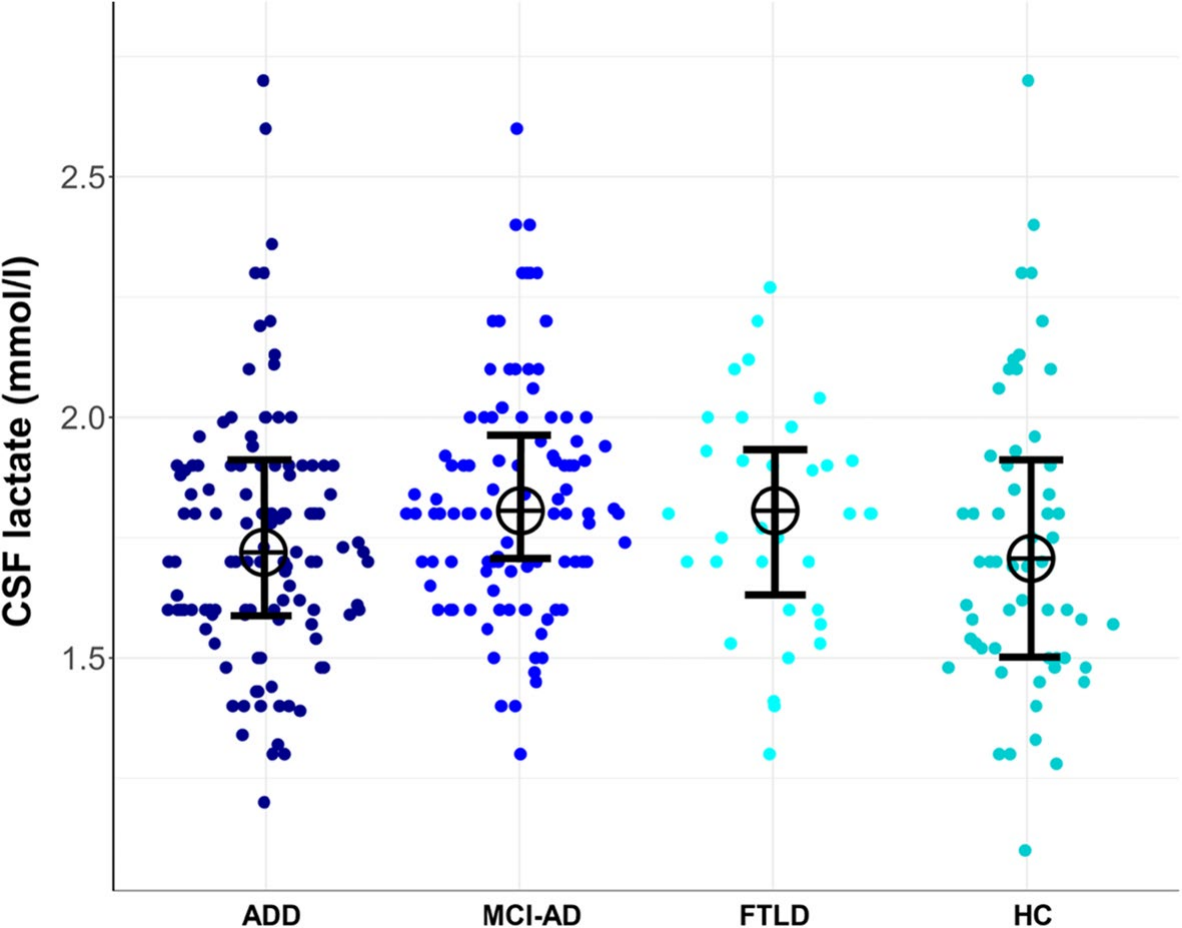
Sina plot of lactate concentrations on individual data level for subsamples. Note: ADD, Alzheimer’s disease dementia; MCI-AD, mild cognitive impairment due to Alzheimer’s disease; FTLD, frontotemporal lobar degeneration; HC, healthy controls. Crosses are medians of lactate concentrations; error bars are interquartile ranges

Figure 2 shows individual level data for lactate concentrations between groups. A Kruskal-Wallis-test revealed significant differences between subsamples in lactate concentrations (*X*^2^ [3] = 10.78, *p* = 0.013). Dwass-Steel-Critchlow-Fligner pairwise comparisons revealed higher values for MCI-AD compared to ADD (*W* = 3.84, *p* = 0.033, *r* = 0.212) and HC (*W* = 3.78, *p* = 0.038, *r* = 0.256).

In an additional analysis, we performed multivariate regression on lactate concentrations with diagnostic group (ADD, MCI-AD, FTLD and HC) as a categorical predictor and—based on above mentioned correlational analyses—age and Alb CSF/S as continuous predictors. Group was dummy-coded (MCI as reference level based on results of the Kruskal-Wallis test and descriptive values of lactate concentrations). The overall model was significant (*F* [5,306] = 6.68, *p* < 0.001), each age (*β* = 0.12, *p* = 0.040) and Alb CSF/S (*β* = 0.23, *p* < 0.001) significantly predicted lactate concentrations. Group category was a significant predictor for ADD vs. MCI-AD (*β* = − 0.33, *p* = 0.013), but failed to reach significance for HC vs. MCI-AD (*β* = − 0.32, *p* = 0.056). After including the interaction of age and Alb CSF/S as a predictor in the regression model, only group category remained significant as a predictor (*F* [6,305] = 6.18, *p* < 0.001; *ß* = 0.35, *p* = 0.008 for ADD vs MCI-AD). Further regression analyses with each ADD, FTLD and HC at the reference level did not provide additional information on categorial predictors. Multivariate regression under consideration of a more rigid exclusion criteria for BBB integrity as mentioned above (Alb-CSF/S > 10) showed comparable results with a significant model (*F* [5,270] = 4.38, *p* < 0.001). Group category (MCI-AD vs. ADD; *β* = 0.34, *p* = 0.016) and Alb CSF/S (*β* = 0.17, *p* = 0.004) significantly predicted lactate concentrations, while age did not *(p* = 0.107).

## Discussion

In this study, we compared CSF lactate levels from a well-defined sample of patients along the AD continuum (MCI-AD and ADD) with HC and patients with FTLD, while taking into account BBB integrity, age, and proxies of disease severity. Across subgroups, we found correlations of CSF lactate with age and Alb CSF/S as a marker of BBB. Neither CSF biomarkers for AD nor MMSE scores were associated with CSF lactate levels for subgroups or in pooled analyses of all patients after adjusting alpha-values for multiple comparisons. Univariate between group comparisons revealed higher CSF lactate levels for MCI-AD compared to ADD and HC. In a multivariate analysis controlling for age and BBB integrity, group remained significant as a categorical predictor of lactate levels when comparing MCI-AD and ADD.

These results are in contrast to the few existing studies on this topic, which predominantly found higher CSF lactate levels in patients with ADD compared to healthy controls [11–14]. As outlined above, most of these studies were characterized by small sample sizes and limited biological and clinical characterization of AD patients and control subjects. Nowadays, AD is being conceptualized in a biologically defined framework and study inclusion and exclusion criteria should rely on CSF or imaging biomarkers [16]. Our study not only provides data regarding CSF lactate levels in a well-defined sample across the spectrum of AD, but also takes into account potential influencing factors like age, BBB integrity, and clinical and biological proxies for disease severity. While we could not confirm higher CSF lactate levels in patients with ADD compared to HC, nor in patients with MCI-AD or FTLD, we observed a significant elevation of CSF lactate in patients with MCI-AD compared to those with ADD. Interestingly, data from Liguori et al.’s sample (2015) also points to a non-linear relationship between AD severity and CSF lactate levels, as they found higher levels in patients with ‘mild AD’ (defined as an MMSE-score above 20) compared to patients with ‘moderate to severe AD’ (below 21). We speculate that this could reflect microglial activation in the early disease stages of AD, where these could play a particularly important role [32, 33]. Latent infectious processes could also contribute, with the infectious agents retained by amyloid beta in later disease stages as amyloid has anti-infectious properties [34, 35]. This hypothesis would be supported by an inverse correlation of Aβ42 and CSF lactate levels in ADD, which was numerically present but, at least in our sample, not statistically significant.

The correlation of age and CSF lactate levels is in accordance with data from a large and clinically diverse sample [6]. Furthermore, we observed a correlation of BBB integrity and CSF lactate in a pooled analysis across subgroups. Disturbances of BBB function might affect lactate efflux and influx regulation, and dysfunction of the BBB has been linked to aging processes in the CNS [17] and is considered a possible feature of AD [18, 19].

CSF lactate levels were lower in the ADD group compared to the MCI-AD group in our sample and we did not observe correlations of CSF lactate with CSF biomarkers of AD or cognitive scores. This speaks against a linear association of CSF lactate levels and disease severity in AD and stands in contrast to the previous finding of an inverse correlation of t-Tau and CSF lactate in patients with ADD described by Liguori and colleagues (2015).

Several pre-analytical and physiological factors regarding CSF lactate levels should be taken into account that are of relevance when interpreting our results and previous studies. Lactate levels in body fluid samples should not be considered stable over time and are sensitive to temperature [36]. While we followed standardized pre-analytical procedures across subsamples, most other studies examining CSF lactate levels in AD and healthy controls did not provide methodological details in this regard. This might in part explain the divergent study results.

### Limitations

To apply uniform diagnostic criteria to the whole group of AD patients in this retrospective study, AD was diagnosed using the McKhann et al. [29] and not the recently proposed Jack et al. [16] research criteria, which require p181-Tau as a more specific biomarker of tau pathology for a CSF-based diagnosis of AD. Hence, including measures of CSF p181-Tau would have increased the diagnostic certainty of AD patients in our study.

Furthermore, lactate not only originates in the CNS, but also in the periphery. As mentioned above, lactate crosses the BBB. Hence, CSF lactate levels should be interpreted cautiously when being understood as a proxy for metabolic processes in the CNS. Impaired regulatory mechanism of the BBB might account for variance. For this reason, peripheral blood lactate levels should be considered in future studies when examining CNS-related measurements of lactate. Also, associations of CSF lactate and additional CSF and peripheral markers of AD and neuroinflammation should be considered in future studies to further elucidate the relationship of AD and inflammatory processes in clinical samples.

## Conclusions

In summary, CSF lactate is not indicative of biological or clinical disease severity in AD, nor does it differentiate between patients with ADD and healthy controls or patients with FTLD. Interestingly, our results point to higher CSF lactate levels in earlier stages of AD, which might be interpreted in the context of microglial activation or the infection hypothesis of AD.

## Data Availability

All data supporting our findings can be requested by contacting the authors on reasonable request. However, due to the nature of pseudonymized patient data, a material transfer agreement is required to meet ethical standards and data privacy laws of Germany.
